# Identification of circular RNA expression profiles in renal fibrosis induced by obstructive injury

**DOI:** 10.1080/0886022X.2021.1979040

**Published:** 2021-10-04

**Authors:** Jiangju Huang, Zhihao Zhang, Benquan Liu, Ying Gao, Jiayi Nie, Shihong Wen, Xiaohong Lai, Hua Liang

**Affiliations:** a Department of Anesthesiology, The First People’s Hospital of Foshan, Foshan, China; b Department of Anesthesiology, The First Hospital of ChangSha, ChangSha, China; c Department of Anesthesiology, The First Affiliated Hospital of Sun Yat-Sen University, Guangzhou, China

**Keywords:** Renal fibrosis, UUO, circRNAs, ceRNA, bioinformatics analysis

## Abstract

**Introduction:**

Advancing renal fibrosis is the common histopathological feature of chronic obstructive nephropathy, representing the final pathway of nearly all chronic and progressive nephropathies. Increasing evidences suggest that circular RNAs (circRNAs) are crucial regulatory molecules present at virtually every level of the cellular pathophysiological process. Nonetheless, there are a few evidences for the role of circRNAs in renal fibrosis induced by obstructive nephropathy.

**Aims:**

We performed RNA-seq analysis to analyze the expression profiles of circRNAs in the obstructed kidneys to identify the potential circRNAs and their network.

**Methods:**

With silk ligated the left ureter to establish a mice unilateral ureteral obstruction (UUO) model. Renal tissue circRNAs were obtained and were screened by a circRNA microarray. The circRNA-miRNA-mRNA regulatory network and the target genes were visualized using Cytoscape software.

**Results:**

The microarray results showed that 5454 and 2935 circRNAs were detected in the control and UUO group, respectively. There were 605 circRNAs up-regulated and 745 circRNAs down-regulated in the obstructive kidneys. The top 5 up-regulated and down-regulated circRNAs were chosen for predicting the circRNA/miRNA/target mRNAs triple network. The GO (Gene Ontology) and KEGG (Kyoto Encyclopedia of Genes and Genomes) analysis showed that these circRNAs and the triple network were enriched in the process of apoptosis, p53 signaling pathway, cell growth and cell death, which might participate in the pathogenesis of obstructive nephrology.

**Conclusion:**

Our results show that the dis-regulated circRNAs might play crucial roles in the pathogenesis of obstructive nephropathy, which proceeds to identify novel therapeutic targets for chronic kidney disease.

## 1. Introduction

Chronic kidney disease (CKD) has become a serious global health problem during the most recent decades [1]. Epidemiological published studies reveal that the incidence of CKD is still rising. CKD also has become a big public health issue in China and caused severe long-term effects of devastating personal and societal consequences [2]. Obstructive kidney disease secondary to urinary tract obstruction is a common complication in the clinic setting, often resulting in CKD and end-stage renal disease.

Advancing renal fibrosis is the common histopathological feature of chronic obstructive nephropathy as previously reported. It represents the common final pathway of nearly all chronic and progressive nephropathies [3]. Fibrosis is a pathologic extension of the normal wound healing process with myofibroblasts activation and migration [4]. Although the molecular mechanisms driving the progression of renal fibrosis are better understood from two decades ago, only a few effective therapeutic strategies are in clinical setting use. A deeper understanding of the molecular mechanisms regulating fibrogenic events is extremely needed [5].

As functional RNA molecules, non-coding RNAs are originally transcribed from DNA while not further code for proteins [6]. In the kidney, increasing evidences suggest that non-coding RNAs act as critical players in renal fibrosis [7]. CircRNA, a type of non-coding RNA, functions as a molecular sponge to sequester miRNA molecules and prevent the targeted mRNA [8]. The growing evidence indicates that circRNAs are crucial regulatory molecules present at virtually every level of the cellular pathophysiological process. However, there is little evidence for the role of circRNAs in renal fibrosis induced by obstructive nephropathy. In this study, we performed RNA-seq analysis to analyze the expression profiles of circRNAs in the obstructed kidneys to identify the potential circRNAs and their network.

## 2. Materials and methods

### 2.1. Animals and UUO model

**Figure 1.**
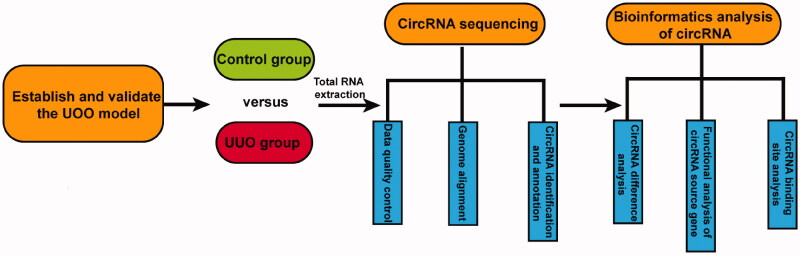
Flowchart of this study.

Animal experiment protocols were approved by the Animal Welfare and Ethics Management Committee of Foshan First People's Hospital (China). All experiments were conducted in accordance with the Guide for the Care and Use of Laboratory Animals. All animals handling and surgical procedures were according to the animal care protocols of Sun Yat-sen University (No. 2017-692) and conformed to the Guidelines of the National Institutes of Health (NIH publication, No. 8023) on the ethical use of animals. C57BL/6 mice (20–30 g, 8 weeks old) were obtained from Guangdong medical laboratory animal center. All animals were housed in plastic cages under a 12 h light/dark cycle with a controlled temperature (22 ± 2 °C) and *ad libitum* access to food and water. All procedures followed the guidelines approved by the Administration Committee of Experimental Animal Care and Use of Sun Yat-Sen University. Mice were divided randomly into two groups. According to the previous report, we established the unilateral ureteral obstruction (UUO) model [9]. Briefly, mice were anesthetized by ketamine and xylazine. *via* a flank incision, the left ureter was exposed and ligated at the ureter-pelvic junction with 4-0 silk. The right kidney was sham-operated and the ureter was not ligated as a control. Kidney tissue was obtained 14 d after UUO surgery [10]. Please see Figure 1 for the flow chart of this study.

### 2.2. Renal morphology

Paraffin-embedded kidney tissue sections were prepared as previously reported. Cut the tissue into 4 μm thick sections. To assess histopathologic changes in the kidney, the sections were stained with H&E (Beyotime Biotechnology, China) and Sirius red (Solarbio life science, American) according to the manufacture’s protocol. Then observed under a light microscope (Olympus, Japan). Classify the pathological abnormalities of the kidney according to the presence and severity of component abnormalities, including fibrosis, glomerulosclerosis, epithelial reactivity, chronic inflammation, vacuolization, necrosis tubular casts, pyknosis, and vascular injury in the sample: 0 = normal kidney (no damage); 1 = minimal damage (<25% damage); 2 = mild damage (25–50% damage); 3 = moderate damage (50–75% damage); and 4 = severe damage (>75% damage) similar as previous described [11]. Sirius red staining was performed to assess the level of renal fibrosis and the content of collagen. Quantitative analysis was performed using NIS-Elements Br 4.0 [12].

### 2.3. Immunofluorescence

Frozen sections of kidneys were cut at 5-μm thickness for ECM proteins staining. Sections were applied with protein blocking solution and then incubated with primary collagen I antibody (ab34710, Abcam, USA), fibronectin antibody (Abcam, USA), and α-SMA antibody (A5228, Sigma, USA). Appropriate secondary antibodies were applied sequentially. Samples in the slides were mounted with 25 μl DAPI for 5 min. Images were visualized using a fluorescence microscope(Olympus Tokyo, Japan) equipped with a digital camera. The average fluorescence intensity(mean) was calculated by using the Image J software. Average fluorescence intensity(mean)=Total fluorescence intensity of this region (INTDEN)/Area of this Area.

### 2.4. Extraction and sequencing of circRNAs

Total RNAs were isolated and purified using TRIzol (Life, cat.265709, CA, USA) following the manufacturer’s procedure. After the quality inspection of Agilent 2100 Bioanalyzer (Agilent, cat.G2939AA, CA, USA) and NanoPhotometer^®^ (Implen, cat.N60, Munich, Germany), ribosomal RNA was removed from 1 μg total RNA using Ribo-off^®^ rRNA Depletion Kit (Human/Mouse/Rat, Vazyme, cat.N406-01, Nanjing, China) and purified using VAHTS^®^ RNA Clean Beads (Vazyme, cat. N412, Nanjing, China). VAHTS^®^ Universal V6 RNA-seq Library Prep Kit for Illumina (Vazyme, cat.NR604-02, Nanjing, China) was used for lncRNA library construction following the manufacturer's protocol. The enriched lncRNAs were segmented with FRAG/PRIME Buffer. The first cDNA strand was generated by reverse transcription starting from −3′, and the second cDNA strand with U was synthesized using the first cDNA strand. Single or dual index adapters were ligated to the fragments with ligase, and size selection was performed with VAHTS^®^ DNA Clean Beads (Vazyme, cat.N411, Nanjing, China). The ligated products were amplified with PCR by the following conditions: Incubated at 37 °C for 10 min to remove the U containing antisense chain, initial denaturation at 98 °C for 10 s; 12–14 cycles of denaturation at 98 °C for 10 s, annealing at 60 °C for 30 s, extension at 72 °C for 30 s, and then final extension at 72 °C for 5 min. The average insert size for the final cDNA library was 290 bp (±50 bp). At last, we performed the 2 × 150bp paired-end sequencing (PE150) on an Illumina Novaseq™ 6000 (Illumina Corporation, San Diego, USA) following the vendor’s recommended protocol by Guangzhou Huayin Health Medical Group CO., Ltd. (Guangzhou, China).

### 2.5. Bioinformatics analysis of circRNA

#### 2.5.1. Data quality control:

The raw reads were processed by removing the adaptor reads and low-quality reads using cutadapt (v1.18) and SOAPnuke (https://github.com/BGI-flexlab/SOAPnuke). rRNA contamination was removed by bowtie2 (https://bowtie-bio.sourceforge.net/bowtie2/index.shtml, v2.2.9) with default parameters.

#### 2.5.2. Genome alignment:

bowtie2 (https://bowtie-bio.sourceforge.net/bowtie2/index.shtml bowtie2, v2.2.9) and Tophat (http://ccb.jhu.edu/software/tophat/index.shtml, v2.1.0) were performed to map clean reads to the reference genome of known species.

#### 2.5.3. CircRNA identification and annotation:

Find_cric (https://github.com/marvin-jens/find_circ,Based on python 2.7) and CIRCexplorer2 (https://circexplorer2.readthedocs.io/en/latest/, v2.3.2) were performed to screen circRNA based on the alignment results. The intersection of the three software was taken as candidate circRNAs. CircRNA was mapped to circBase (http://www.circbase.org) for circRNA annotation.

#### 2.5.4. CircRNA difference analysis:

A self-made program was performed to calculate the expression of circRNA by normalized to TPM (Transcripts per million). The Expdiff method was used to count the different expressions of miRNAs in the two samples. |log2Foldchange| ≥ 1and *p-*value < 0.05 was considered significant.

#### 2.5.5. Functional analysis of circRNA source gene:

GO and KEGG analysis of the source genes was completed by the cluster profile package in R to clarify the function of the significantly different circRNA source genes.

#### 2.5.6. CircRNA binding site analysis:

The miRNA binding sites of candidate circRNAs were using Miranda (http://www.microrna.org/microrna/home.do, v3.3a).

### 2.6. Statistical analysis

All data were presented as mean ± S.E.M. Two groups were conducted using unpaired Student’s *t*-test, *p*-values less than 0.05 were considered statistically significant. KEGG analysis for biological processes with a threshold of FDR ≤ 0.05 and *p*-value < 0.05 was considered statistically significant.

## 3. Results

### 3.1. Construction of the mouse UUO model

**Figure 2.**
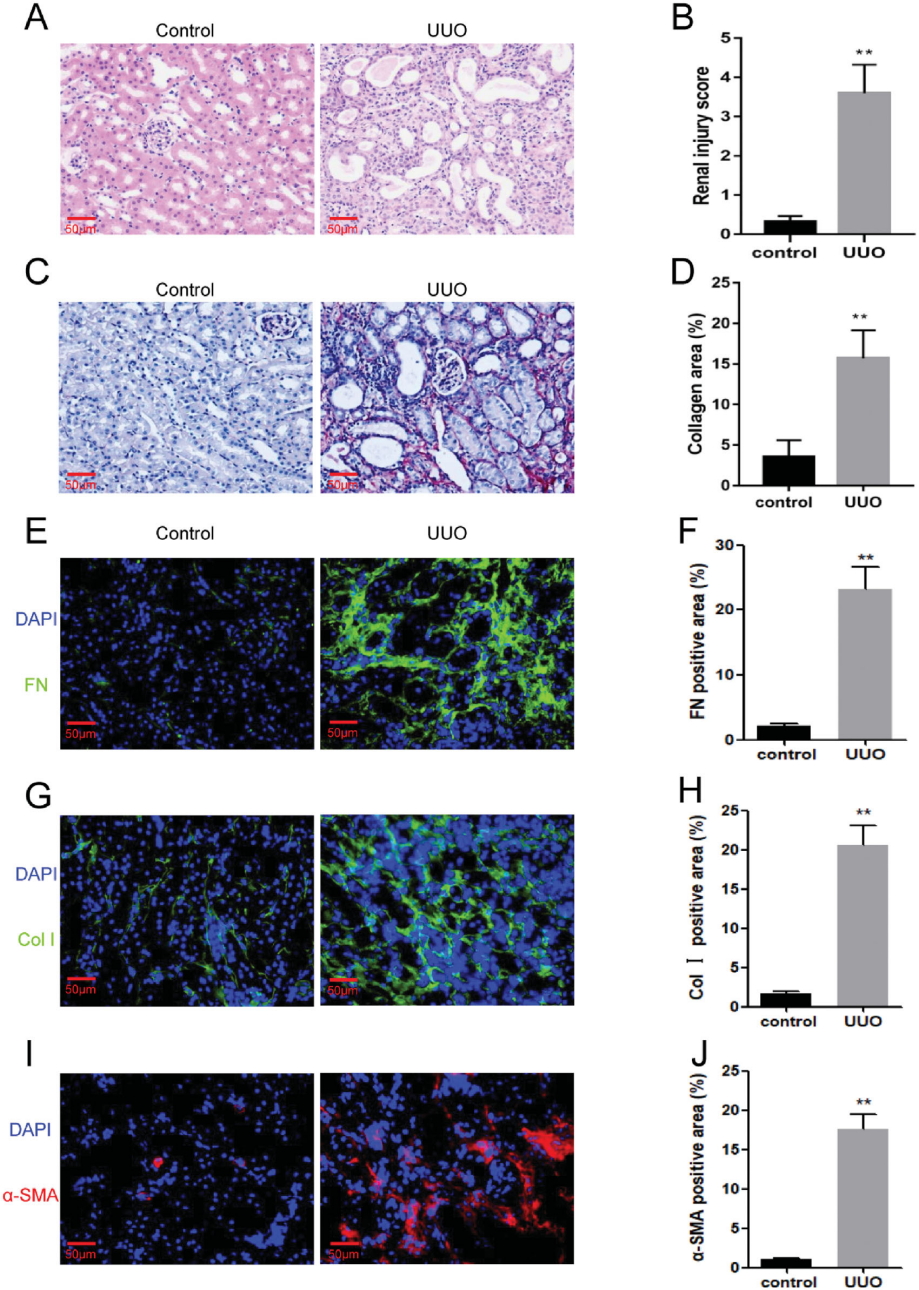
UUO model was conducted successfully. (A) H&E staining for kidneys of UUO mice or control mice. (B) Sirius red staining for kidneys of UUO mice or control mice. (C) Quantitative assessment of renal injury in the kidneys of UUO mice or control mice. (D) Quantitative assessment of collagen areas in the kidneys of UUO mice or control mice. (E) Representative photomicrographs of kidney sections stained for Fibronectin (green) and counterstained with DAPI (blue). (F) Quantitative analysis of the fibronectin-positive area in the kidneys. (G) Representative photomicrographs of kidney sections stained for collagen I (green) and counterstained with DAPI (blue). (H) Quantitative analysis of collagen I-positive area in the kidneys. (I) Representative photomicrographs of kidney sections stained for α smooth muscle actin (α-SMA; red) and counterstained with DAPI (blue). (J) Quantitative analysis of the α-SMA-positive area in the kidneys. **p < 0.01 versus control. *n* = 3 in each group. Scale bar: 50μm.

Fourteen days after the UUO surgery, the mice were sacrificed and the kidney tissue was sampled. Kidney sections from the control and UUO mice were stained with H&E and Sirius red. In the UUO mouse kidney tissues, tubular atrophy and dilation, atrophy or necrosis, and infiltration of inflammatory cells were observed, and the collagen area in the obstructed kidneys of mice was markedly increased as compared to the control kidneys, suggesting robust fibrotic response following UUO (Figure 2(A–D)). In addition, the immunofluorescence results showed that the expression of fibrosis marker genes, fibronectin and collagen I in the obstructive kidneys were significantly up-regulated compared with control mice (Figure 2(E–H)). Furthermore, there was a significant increase of α-SMA positive area in the kidneys of mice following UUO (Figure 2(I–J)). These data demonstrated that the model of UUO was successfully established, which led to severe renal fibrosis. Then, the remaining kidney tissue was tested for circRNA microarray analysis.

### 3.2. Expression profiles of circRNAs in renal fibrosis

**Figure 3.**
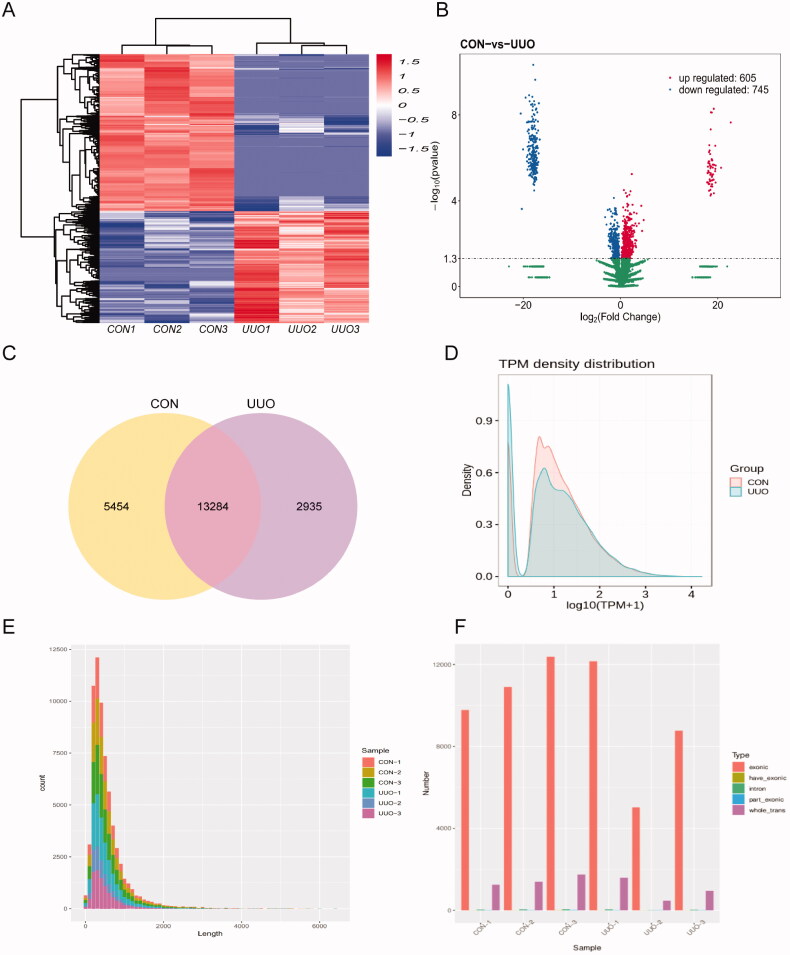
The circRNAs in mouse kidney. (A) Heat map generated by hierarchical clustering of differentially expressed circRNAs in UUO and control samples, red representing high read counts of circRNAs and blue for low expressed circRNAs. (B) The volcano plot shows the total change circRNAs in each group, left shows those down-regulated and right for up-regulated circRNAs. (C) Among detected circRNAs, 5454 and 2935 circRNAs were detected in the control and UUO group, respectively, and 13284 circRNAs were detected in both groups. (D) The TPM distributions of circRNAs. (E) The distribution of the sequence length of circRNAs. (F) The distribution of circRNAs host genes in all chromosomes, most are form exon.

All these DEcircRNAs were displayed in the hierarchical clustering in Figure 3(A), with red and blue colors representing high and low read counts of circRNAs, respectively. The enrichment of total circRNAs in the kidney tissue from UUO mice and control mice is demonstrated in the volcano plot in Figure 3(B). The up-regulated circRNAs were presented as the red dots on the left, while the down-regulated circRNAs were presented as the blue dots on the right. Some of the most increased and mostly decreased circRNAs are listed in supplementary Table 1. It is obvious that a large number of circRNAs were significantly modified by UUO. As shown in Figure 3(C), according to the filtration criteria (fold changes >2.0 and *p* < 0.05), 5454 and 2935 circRNAs were detected in the control and UUO group, respectively, and 13,284 circRNAs were detected in both groups. Transcripts per million reads (TMP) were used to estimate the expression level of the circRNA transcripts. Almost all circRNA transcripts were expressed at low levels (Figure 3(D)). Using a find-circ tool, we found that circRNA transcripts were mostly around 500 bp in length, and source sequences statistical of these circRNAs are shown in Figure 3(E). In addition, we found the number of circRNAs mainly from exonic (Figure 3(F)).

### 3.3. The feature and the potential function of DEcircRNAs

**Figure 4.**
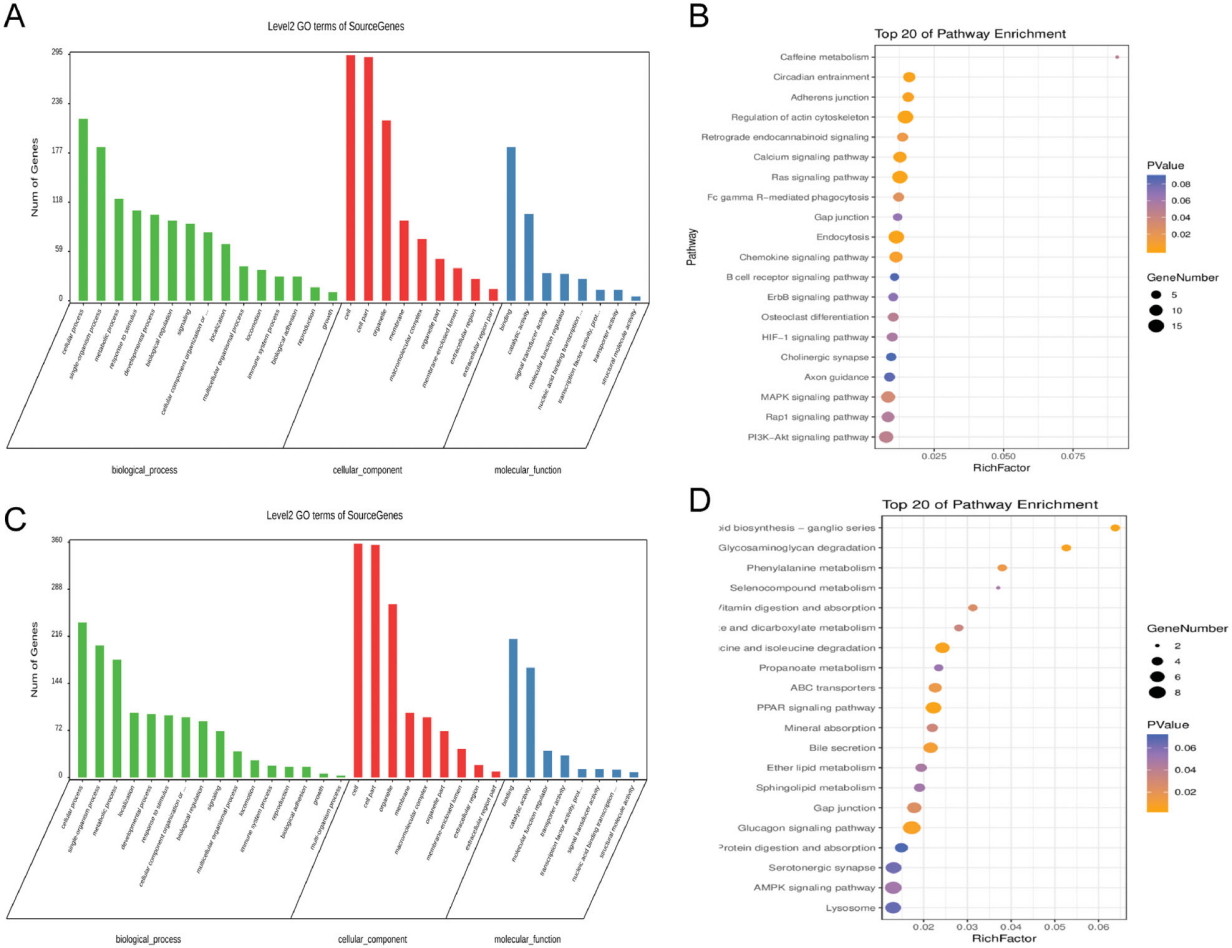
The features of those DEcircRNAs. (A and B) The results of GO and KEGG pathway analysis for upregulated circRNAs. (C and D) The results of GO and KEGG pathway analysis for downregulated circRNAs.

To investigate the host genes of these DEcircRNAs, GO and KEGG analyses were conducted as mentioned above. To investigate the potential function of the DEcircRNAs, we performed functional enrichment analysis based on gene ontology (GO) and KEEG analysis. The result of GO analysis for the up and down-regulated circRNAs respectively was listed in Figure 4(A,C), consisting of three different aspects named biological process (BP), cellular component (CC) and molecular function (MF). According to the KEGG analysis, results of the up-regulated circRNAs, the top 20 most significantly enriched pathways, sulfur metabolism, apoptosis, p53 signaling pathways are presented in Figure 4(B). Besides *p*<0.05, KEGG analysis results of the down-regulated circRNAs are shown in Figure 4(D). The top 20 most significantly enriched pathways include the peroxisome proliferators-activated receptors (PPARs) signaling pathway, Gap junction and Glucagon signaling pathway. And among those the most significantly enriched pathway was the Glucagon signaling pathway (*p*<0.05).

### 3.4. CeRNA analysis for the validated DEcircRNAs

**Figure 5.**
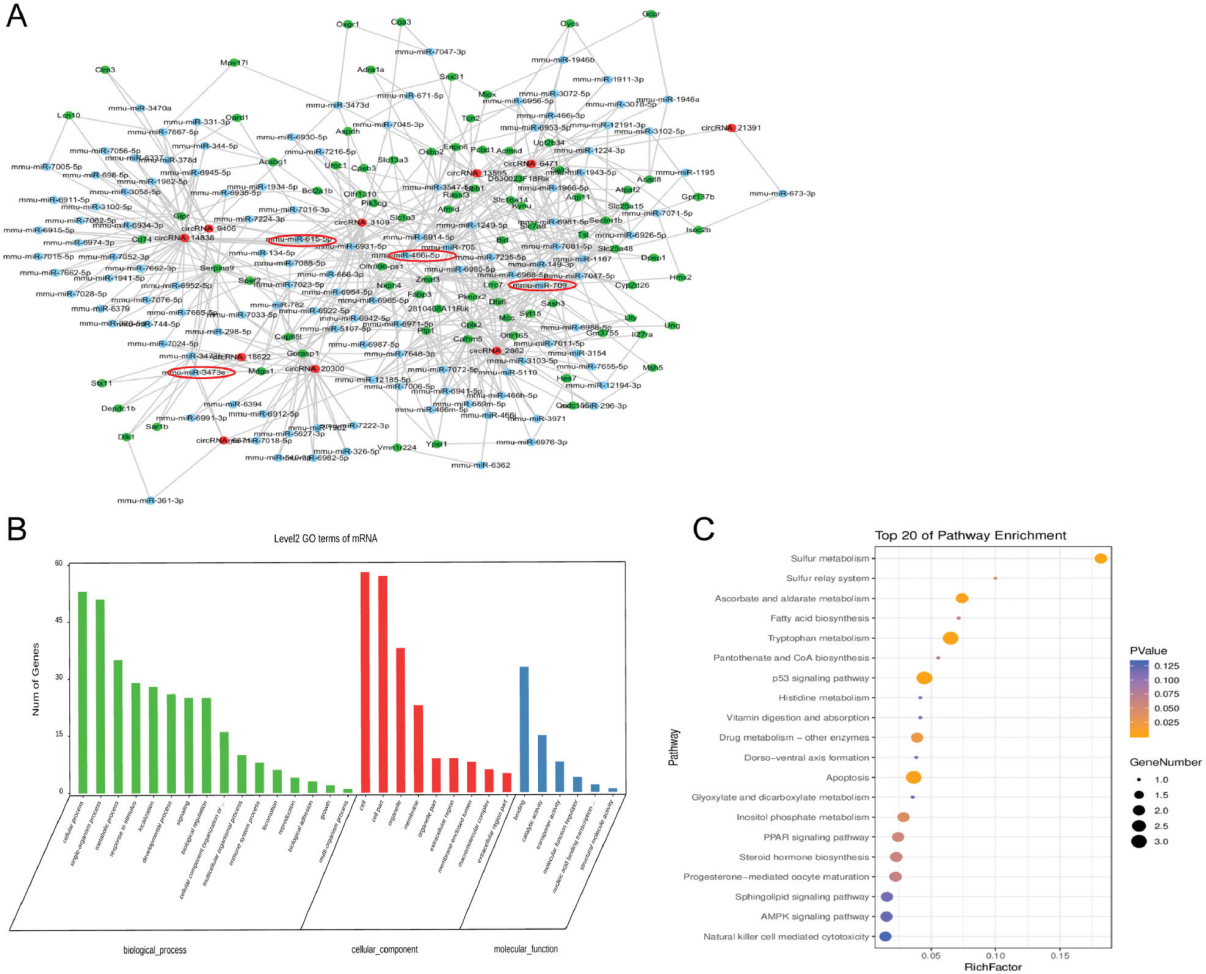
The CeRNA network for the top 10 DEcircRNAs. (A) The ceRNA module network of the top 5 up and downregulated circRNAs; red spots represented circRNAs, blue for miRNAs, green for genes. The high-light red circles were the main ones to present those circRNAs that have been provided to be important in fibrosis renal progress. (B) Gene ontology (GO) analysis of dysregulated mRNAs in the triple network. (C) KEGG pathway shows the top 20 enrichment terms of biological processes.

For penetrating investigating the potential function of the top five up and down circRNAs, we performed coding-noncoding gene co-expression analysis. All of those circRNAs predicted interacting miRNAs and those mRNA were calculated and visualized as the ceRNA network (Figure 5(A)). By mapping these circRNAs into a ceRNA network, we found that these circRNAs and their neighbors formed a complex module. The high-light red circles were the main ones to present those circRNAs that have been provided to be important in fibrosis renal progress. Further, all mRNAs in the module were enriched for the GO analysis, the results indicated that the target mRNAs of the top five up and down circRNAs enriched in the process of apoptosis, p53 signaling pathway, cell growth and death. It indicated that those pathways might mediate the progress of UUO (Figure 5(B)). Therefore, those most 5 up and down changed circRNAs have a potential function in obstructive nephropathy, further studies are required to confirm the detailed effect and the mechanism of these circRNAs.

## 4. Discussion

In this study, we revealed circRNAs profiles characters in the obstructed kidney of mice. Total 605 circRNAs are up-regulated and 745 circRNAs are down-regulated in the kidneys after UUO surgery. And the potential downstream miRNA network of the DEcircRNAs is checked based on the circRNA-miRNA interaction prediction. The GO and KEGG analysis according to the host gene of all microarray-screened DEcircRNA and the genes under putative modulation by validated circRNAs *via* circRNAs/miRNA/mRNA pathways provided an overview for the role circRNAs in the obstructive nephropathy.

Based on the previous reports, the UUO model was constructed by ligating the ureter to induce tubular injury and severe renal fibrosis. The UUO model is widely used to induce the disorder of chronic obstructive nephropathy [13]. The obstructive nephropathy is featured by inflammatory cells infiltration and cytokines release, which causes fibroblasts and myofibroblasts, resulting in extracellular matrix deposition and uncontrolled apoptosis of tubular cells. The pathology in UUO is highly reproducible from one experiment to another [14]. In this study, the obstructive kidneys exhibit typical renal morphology in H&E staining slides. Moreover, our results reveal that the expression of extracellular matrix (ECM) proteins, the marker proteins of myofibroblasts and the collagen area in the kidneys of UUO mice were markedly increased. These data show convincing evidence that the model of renal fibrosis induced by obstructive nephropathy was successfully established in the present study.

Renal fibrosis mechanisms are complicated involve a large number of signaling molecules, a multitude of pathways [15]. Mounting evidences have demonstrated that apoptosis contributes to the pathogenesis of renal fibrosis and related kidney diseases [16]. Autophagy, a cellular process of degradation of damaged cytoplasmic components and regulates cell death and proliferation, plays a protective role in the development of renal fibrosis [17]. Moreover, there is evidence that p53 and its family members may also contribute to acute kidney injury and the fibrotic process [18]. Our results of KEGG analysis show that the most significantly enriched pathways include apoptosis, natural killer cell-mediated cytotoxicity, adherence junction, calcium signaling pathway, mitogen-activated protein kinase (MAPK) signaling pathway, PI3K-Akt signaling pathway, endocytosis, and Ras signaling pathway in the up-regulated circRNAs. Whereas, the down-regulated circRNAs contain PPARs signaling pathway, Gap junction and Glucagon signaling pathway. Our enrichment analysis of gene ontology for the target mRNAs indicates that the predicted target mRNAs also are enriched in the process of apoptosis, p53 signaling pathway, cell growth and cell death.

Most circRNAs are produced from known protein-coding genes and consist of a single exon or multiple exons [19]. Recent progress in circRNA research reveals that circRNA is important in biology and pathobiology [20], To date, circRNAs have been implicated in many human diseases, including immune responses and immune diseases [21], cancer and Alzheimer’s Disease [22]. Recently, there is a lot of evidence shows that circRNA participates in the pathogenesis of certain renal diseases, such as renal cell carcinoma, acute kidney injury, diabetic nephropathy and lupus nephritis, and may even serve as a biomarker in kidney diseases. Whereas, the feature and function of circRNAs in renal diseases remain ambiguous [23]. Nevertheless, there is no evidence that whether circRNAs participate in the process of renal fibrosis induced by obstructive nephropathy.

It was reported that circRNA may function as a sponge of miRNAs to regulate the downstream gene expression [24]. To further demonstrate the effects of circRNAs on renal fibrosis induced by obstructive nephropathy, we used circRNA-miRNA-mRNA interaction data to construct a triple ceRNA network. Our results reveal that 605 overexpressed circRNAs and 745 low-expressed circRNAs are found in the obstructed kidneys, and a triple ceRNA network about circRNA-miRNA-mRNA interaction data is constructed.

There is evidence that miRNA is considered to be a negative regulator of the renal fibrotic pathway. MiRNA-299a-5p is upregulated in the mice kidney and miRNA-299a-5p inhibition protects these mice against renal fibrosis and CKD severity [25]. In the focal segmental glomerulosclerosis model, miR-615-5p is down-regulated and negatively correlated with the degree of podocyte damage and renal fibrosis, targeting the downstream of collagen I and transforming growth factor-β1 to participate in the pathological process of focal segmental glomerular sclerosis (FSGS) [26]. The expression level of miR-709 in the renal proximal tubular cells of patients with AKI correlated with the severity of kidney injury reveals a pathogenic role in tubular injury [27]. The miRNA-3473 is specifically up-regulated in the glomerular injury models, may become one of the early and sensitive indicators to detect tubular and glomerular injuries [28]. The miR-466i and miR-466k are sponged by circRNA-Kcnq1ot1 participate in cardiomyocytes apoptosis in acute myocardial infarction [29]. By analyzing the ceRNA results, we reveal that the up-regulated circRNA-3109 or circRNA-14838 is able to suppress miR-615-5p, which binds to collagen I, may promote renal fibrosis [30]. Furthermore, it is reasonable to assume that the dysregulated circRNA-2862 may sponge with miRNA-709, circRNA-6671 with miRNA-3473, circRNA-6471 with miRNA-466 and relieve their suppression to downstream target may participate in the process of kidney injured by UUO. The circRNA ceRNA network in mouse kidney tissue is complex and furthermore investigation needs to be clarified for their potential function in obstructive nephropathy.

There are still some limitations in this study. We analyzed the difference of circRNAs in kidney tissue between normal mice and UUO mice, and the sample size of each group in this study was 3. Usually, *N* = 3 is small sample size for animal experiments. However, we examined the inter-sample expression correlation for intra-group sample differences (Pearson correlation). Pearson correlation coefficients of the control group were all above 0.65, and Pearson correlation coefficients in UUO were all above 0.5, indicating that there was little difference within the group and the sample size was acceptable. The ultimate purpose of this study is to provide some ideas for the diagnosis and treatment of chronic kidney injury in clinical practice. The analysis of these results came from the mice model, we will conduct homology analysis on these circRNAs, find out the mouse circRNAs corresponding to human circRNAs, and further study the latter. It is hoped to provide a feasible target for the diagnosis and treatment of chronic kidney injury in the future.

## 5. Conclusions

In conclusion, this is the first study to systematically analyze circRNAs and related-ceRNA networks in obstructive nephropathy. We have characterized a profile of dysregulated circRNAs that might be prospective clinical markers associated with the development of obstructive nephropathy. However, based on these results, future work is needed to uncover the underlying molecular mechanisms of circRNAs in renal fibrosis induced by obstructive injury.

## Author contributions

Jiangju Huang and Zhihao Zhang contributed equally to this work.

## Supplementary Material

Supplemental Material

Supplemental Material

Supplemental Material

Supplemental Material

## Data Availability

The data used to support the findings of this study are available from the corresponding author upon request.

## Abbreviations

UUO: unilateral ureteral obstruction
CKD: chronic kidney disease
ceRNA: competing endogenous RNA
CircRNA: circular RNA
miRNA: microRNA
DEcircRNAs: Differentially expressed circRNAs
KEGG: Kyoto Encyclopedia of Genes and Genomes
GO: The Gene Ontology
PPARs: peroxisome proliferators-activated receptors
FSGS: focal segmental glomurular sclerosis
MAPK: mitogen-activated protein kinase
